# Impact of Mono-, Di-, and Trivalent Ions on the Rheology of Borate-Crosslinked Guar Fracturing Fluids

**DOI:** 10.3390/gels12050373

**Published:** 2026-04-29

**Authors:** Boyang Liu, Zhenhua Li, Lianguo Wang, Chenhao Li, Ya Wu, Yongfei Li, Dan Zhao, Gang Chen, Weiyu Bi

**Affiliations:** 1 Engineering Research Center of Oil and Gas Field Chemistry, Universities of Shaanxi Province, Xi’an Shiyou University, Xi’an 710065, China; 23211071009@stumail.xsyu.edu.cn (B.L.); 23211071007@stumail.xsyu.edu.cn (Z.L.); yfli@xsyu.edu.cn (Y.L.); gangchen@xsyu.edu.cn (G.C.); 2 The 11th Oil Extraction Factory, Changqing Oilfield Company (PetroChina), Qingyang 745000, China; wlg_cq@petrochina.com.cn; 3 College of Petroleum Engineering, China University of Petroleum (Beijing), Beijing 102249, China; 2025310164@student.cup.edu.cn (C.L.); 2024310160@student.cup.edu.cn (D.Z.); 4 Shaanxi Province Key Laboratory of Environmental Pollution Control and Reservoir Protection Technology of Oilfields, Xi’an Shiyou University, Xi’an 710065, China; 5 Oil and Gas Technology Research Institute, Changqing Oilfield Company (PetroChina), Xi’an 710021, China

**Keywords:** salt ions, differential effect, viscosity, viscoelasticity, fracturing fluids

## Abstract

Water-based fracturing fluids, which are essential for enhancing oil and gas production, increasingly utilize seawater or produced water as alternatives to freshwater due to scarcity and cost considerations. However, the high salinity of these alternative water sources can compromise fluid stability and induce formation damage. Herein, the rheological behavior of borate-crosslinked hydroxypropyl guar (HPG) fracturing fluids was systematically evaluated in the presence of individual salts to elucidate the effects of ionic composition and concentration. Viscosity measurements at 80 °C and 170 s^−1^ revealed that Ca^2+^ above 1500 mg/L reduced viscosity to below 50 mPa·s within 50 min, whereas Na^+^, K^+^, Mg^2+^ and SO_4_^2−^ up to 10,000 mg/L exhibited no significant influence on viscosity and shear resistance. Among the cations investigated, Fe^3+^ exerted the most severe effect: only 15 mg/L Fe^3+^ caused viscosity to drop below 50 mPa·s within 30 min, far below the requirement for field applications. At elevated concentrations, MgCl_2_, CaCl_2_ and FeCl_3_ compromised gel structural strength, while KCl-containing fluids demonstrated superior elastic resistance compared to NaCl at equivalent high concentrations. Microstructural analysis by SEM revealed that Na^+^, K^+^ and Mg^2+^ enhanced polymer hydration and HPG fiber entanglement, promoting the formation of well-defined network structures. In contrast, Ca^2+^ and Fe^3+^ disrupted the crosslinked gel architecture through complexation and electrostatic interactions with the polymer, resulting in reduced structural integrity. These findings provide critical insights for formulating fracturing fluids using saline or recycled water sources and inform targeted pretreatment strategies for flowback water in hydraulic fracturing operations.

## 1. Introduction

Hydraulic fracturing is widely used to improve oil and gas production by increasing reservoir permeability and improving productivity [1]. Hydraulic fracturing operations are known to be water-intensive, typically requiring 10,000 to 50,000 cubic meters of freshwater per well for conventional and shale reservoir stimulations. Such large-scale freshwater consumption places significant pressure on local water resources, especially in water-scarce oil and gas regions [2,3,4]. Due to the cost and scarcity of freshwater and the logistical difficulties in transporting it, water sources such as flowback water, seawater, and produced water could potentially be applied to replace freshwater in hydraulic fracturing operations [5]. Saline water has been the subject of extensive research to reduce freshwater consumption [6,7].

The utilization of saline water in hydraulic fracturing presents unique challenges. High salinity levels can change the rheological and viscoelastic properties of polymer chains in fracturing fluids, thereby impacting fluid stability and potentially causing formation damage [8]. For example, Zeng et al. systematically investigated fluid–shale interactions under high salinity conditions and clarified that salt–fluid interactions dominated the salinity evolution of flowback water during shale fracturing [9]. Gupta and co-workers further demonstrated that high concentrations of Ca^2+^ and Mg^2+^ significantly reduced the thermal stability and apparent viscosity of fracturing fluids, thereby impairing their overall performance under reservoir conditions [10]. Similarly, Gaurina-Međimurec’s group found that both monovalent ions (Na^+^ and K^+^) and divalent ions (Ca^2+^ and Mg^2+^) affected the viscosity and stability of carboxymethyl HPG gels, with monovalent ions reducing viscosity only in the absence of divalent ions [11]. Othman’s research indicated that the concentration of salt had minimal impact on the rheology of carboxymethyl HPG polymers, except for Na_2_SO_4_. They also observed that at lower temperatures, low concentrations of CaCl_2_ resulted in increased viscosities of mixing viscoelastic surfactants and carboxymethyl cellulose fluids [12]. Salts can alter the conformation of polymers, affect the interaction of crosslinking agents, or delay or prevent polymer hydration, all of which can impact the viscoelastic properties of fracturing fluids [13]. The influence of the composition and content of saline water on fluid blending has become an important reference index for performance evaluation and application of hydraulic fracturing fluid [14,15]. An understanding of these factors is essential for optimizing the use of saline water in hydraulic fracturing operations and ensuring the effectiveness of the fracturing processes.

HPG fracturing fluid is one of the most practical and feasible hydraulic fracturing fluid technologies because of its low price and little residue content after gel breaking, which is usually used for enhanced exploitation of low- and ultra-low-permeability reservoirs [16,17,18]. HPG is used in fracturing fluids as a thickener, which consists of a polymannose backbone with galactose branches [19]. However, when used alone, HPG does not generate sufficient viscosity to effectively carry and deliver a proppant during the hydraulic fracturing process. To overcome this limitation, a crosslinker is employed to interact with the HPG molecules, forming gels and achieving high viscosity values [20].

Borate is the most popular compound for crosslinking guar-based fracturing fluids. The cis-vicinal dihydroxyl groups of guar gum polysaccharides form viscoelastic gels through physical and chemical interactions with boron crosslinkers [21]. The crosslinking reaction between borate and HPG is usually reversible, which means it can undergo reversible dissociation and exhibit good shear resistance [22]. Despite its advantages, the crosslinking between HPG and either borate or metal ions exhibits variations. The borate-crosslinked HPG chains are susceptible to physical breakage [23]; however, the salt effects of HPG gels in complicated solutions containing borate and metal ions are unknown.

Several mechanisms have been suggested to elucidate the controlling factors behind the high salinity [24,25,26,27]. By examining the impact of individual ions on polymer hydration and rheology, a better understanding of the intricate effects of multiple metal ions in saline water can be achieved, thereby enabling the development of strategies to enhance the rheology of fracturing fluids. However, water composition and salinity vary from region to region, and the composition of fracturing fluids, including their thickeners and crosslinkers, differs among oil fields [1]. For example, the influence of Fe^3+^ generated by corrosion in flowback water cannot be neglected [28]. There has been limited comprehensive, in-depth discussion on the effects of individual salts on the crosslinking interactions between HPG polymer fluids and salts, with even less attention given to the interactions between trivalent ions and HPG polymers. To improve the temperature and shear resistance of fracturing fluids prepared with saline or reused water, the effects of inorganic salt ions on the shear properties, storage modulus, and loss modulus of HPG polymer were investigated based on the composition and properties of formation water and flowback water used in hydraulic fracturing. The microstructure of the interaction between inorganic salt ions and HPG was analyzed. The effects and mechanisms of action of ion types (Na^+^, K^+^, Ca^2+^, Mg^2+^, Fe^3+,^ and SO_4_^2−^) and concentrations on the gelation performance of fracturing fluid were discussed, and the microscopic mechanisms by which ions affect the gelation process of fracturing fluid were revealed.

This study deepens the understanding of ion-induced gelation behavior of HPG fracturing fluids and lays a theoretical foundation for further research on HPG fracturing fluids prepared with saline water. It provides important theoretical support for exploring methods to optimize fracturing fluids prepared with saline water or flowback water, as well as for developing targeted treatment measures for fracturing fluids.

## 2. Results and Discussion

### 2.1. The Pick-Up and Suspension Capabilities of Fracturing Fluids with Varied Salt Concentrations

**Figure 1:**
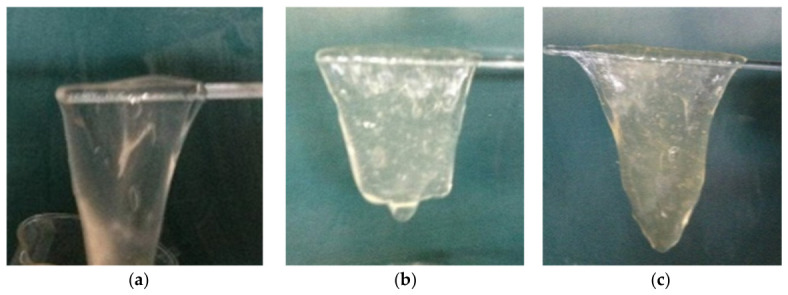

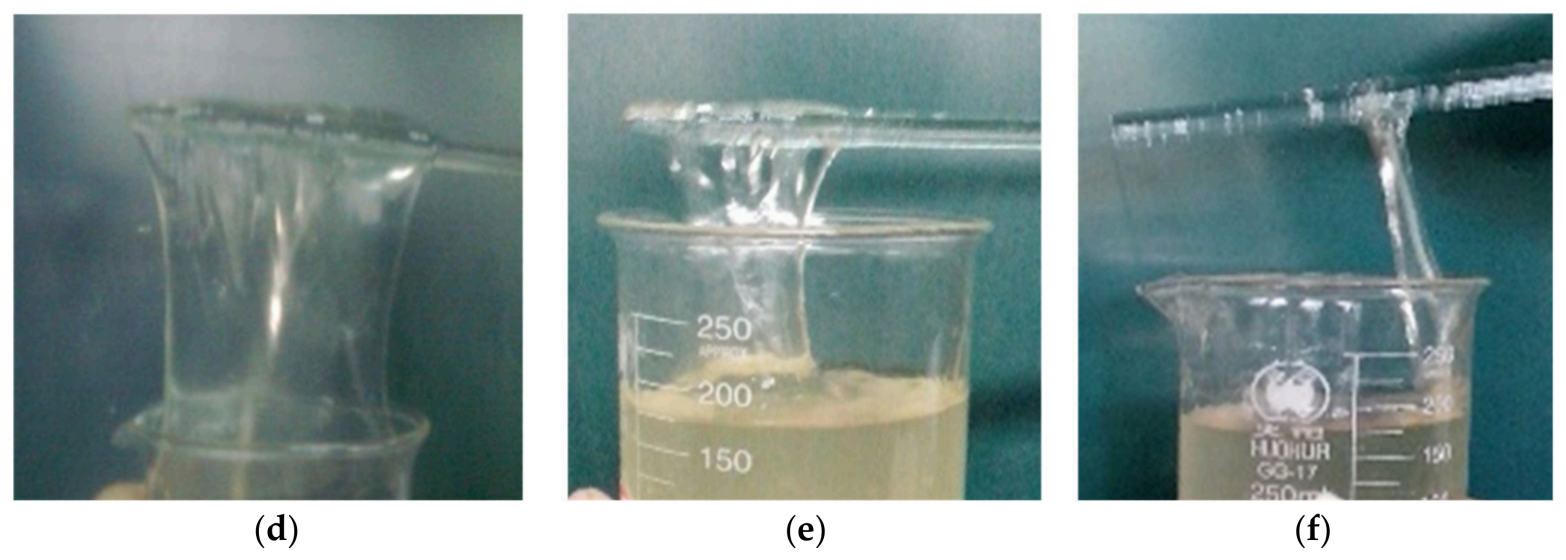
Polymeric films of (**a**) 10,000 mg/L NaCl, (**b**) 10,000 mg/L KCl, (**c**) 2000 mg/L Na_2_SO_4_, (**d**) 2000 mg/L MgCl_2_, (**e**) 2000 mg/L CaCl_2_, (**f**) 30 mg/L FeCl_3_.

The pick-up and suspension capabilities of fracturing fluids are one of their critical performance indicators, and this performance may fluctuate with varied salt concentrations. Taking into account the composition and content of formation water in northwestern China, Figure 1 demonstrates how different salt concentration environments affect the pick-up and suspension capabilities of HPG fracturing fluids. In fluids containing 10,000 mg/L of NaCl and KCl, the HPG fracturing fluid exhibited a higher level of crosslinking, resulting in superior pick-up and suspension performance. Likewise, fluids with 2000 mg/L of Na_2_SO_4_ and MgCl_2_ also demonstrated good pick-up and suspension performance. Conversely, in fluids containing 2000 mg/L of CaCl_2_ and FeCl_3_, the crosslinking degree of the HPG fracturing fluid decreased, causing a decline in the pick-up and suspension capabilities. It is imperative to conduct thorough research on the rheological properties of hydroxypropyl guar gum fracturing fluids and their influencing factors, and subsequently to optimize formulations and construction techniques based on these findings.

### 2.2. Salts’ Effect on Viscosity of Fracturing Fluids

#### 2.2.1. Effect of Monovalent Ions

**Figure 2:**
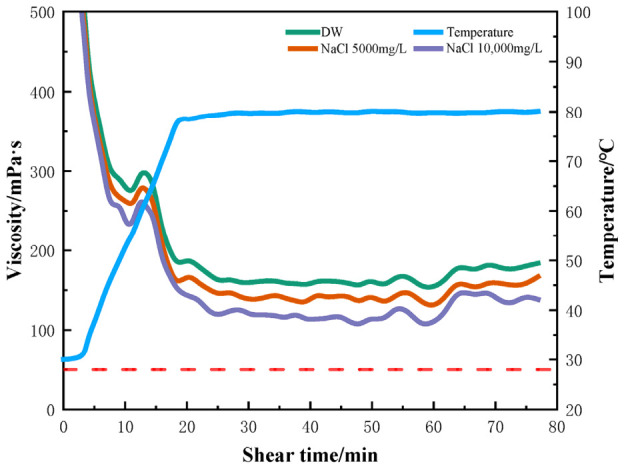
Rheology of HPG fracturing fluid prepared with different concentrations of NaCl.

**Figure 3:**
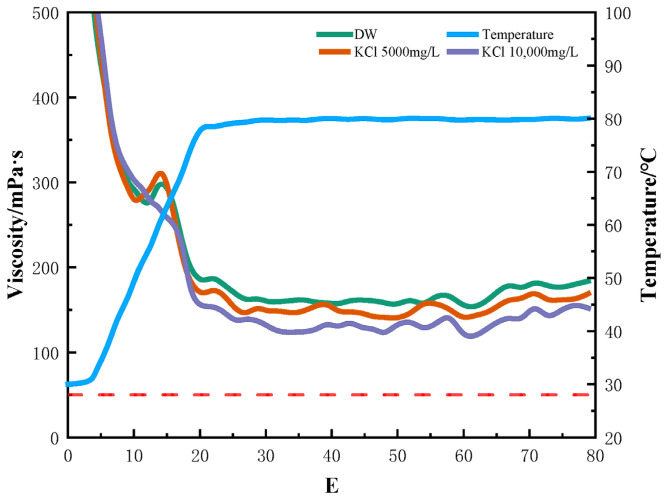
Rheology of HPG fracturing fluid prepared with different concentrations of KCl.

Saline water, such as seawater, produced water, and formation water with high salinity, has lately become a popular alternative to freshwater [29]. However, high salinity can change the rheology and viscoelasticity of polymer chains of fracturing fluids and thus can impact the fluid stability, resulting in formation damage [30]. To achieve a comprehensive understanding of the complex effects of multi-metal ions in seawater, the effects of salt type and concentration on the performance of the fracturing fluids were systematically examined, using only individual salts. NaCl and KCl are the most common inorganic salts in formation water and reused water [31]. The effects of monovalent ions on the viscosity of polymer fluids were investigated first, and the rheological profiles of crosslinked gels in NaCl and KCl solutions are shown in Figure 2 and Figure 3. It can be seen from Figure 2 and Figure 3 that compared with the crosslinked HPG gel in deionized water, the apparent viscosity of HPG gel in NaCl and KCl solutions did not change significantly. Even when the concentration of NaCl or KCl was higher than 10,000 mg/L, the viscosity did not decrease and remained above 100 mPa·s at 80 °C after shearing for 70 min, indicating that monovalent (Na^+^ and K^+^) ions have little effect on the viscosity and stability of fracturing fluids. In the presence of Na^+^ and K^+,^ the covalent and hydrogen bonding between molecules was partly disrupted due to the ionic charge, the curled molecules stretched out, and hydration was enhanced; the shear viscosity was not reduced and even slightly increased [22].

**Figure 4:**
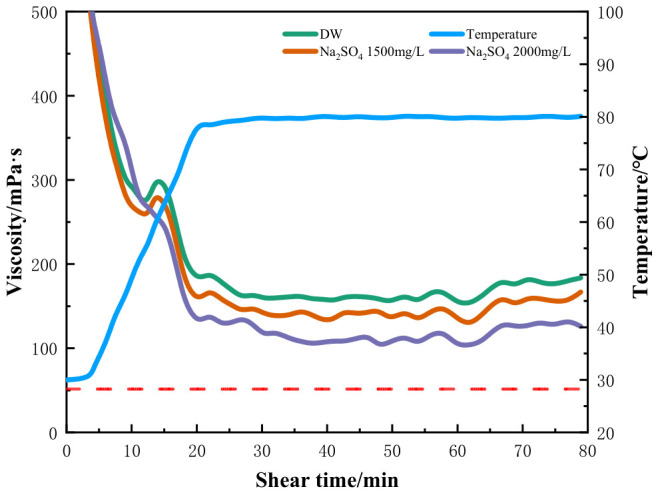
Rheology of HPG fracturing fluid prepared with different concentrations of Na_2_SO_4_.

To compare the anion effect, the influence of Na_2_SO_4_ on the rheological properties of the fracturing fluids was further investigated. Figure 4 shows that the Na_2_SO_4_ concentration in the formulation water up to 2000 mg/L also has little effect on the performance of the crosslinked HPG gel [32]. It can be concluded that the hydration of cations is stronger than that of anions, and the effect of cations on the temperature and shear resistance properties of fracturing fluids is greater than that of anions [33].

#### 2.2.2. Effect of Divalent Inorganic Salts on the Temperature and Shear Resistance of Fracturing Fluids

**Figure 5:**
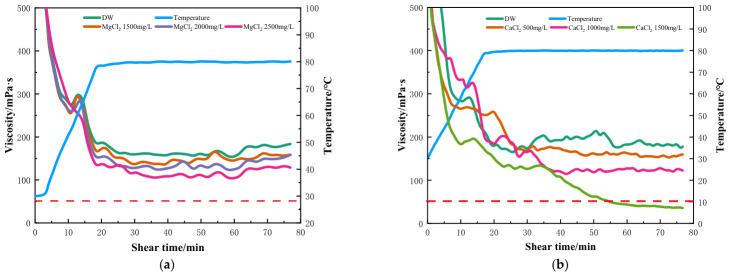
Rheological properties of HPG fracturing fluid with different concentrations of inorganic salt solutions. (**a**) MgCl_2_; (**b**) CaCl_2_.

Divalent Ca^2+^ and Mg^2+^ ions in produced water and seawater usually exist in high concentrations [34], thus the effects of MgCl_2_ and CaCl_2_ on the rheology of HPG fluids were further investigated. The results are shown in Figure 5a,b. The profiles of Figure 5a show that the shear viscosity slightly decreased even when a high concentration of MgCl_2_ was added to the HPG fluid. For instance, when the MgCl_2_ concentration increased to 2500 mg/L, the viscosity was still greater than 100 mPa·s when sheared at 80 °C for more than 70 min. Different from the literature reports indicating that Mg^2+^ ions reduce the viscosity of water-based fracturing fluids, the crosslinked HPG gel, including high concentrations of Mg^2+^ can still meet the requirements of fracturing jobs. Unexpectedly, MgCl_2_ had little effect on the performance of the fracturing fluid within a concentration of 2500 mg/L.

In contrast, with the increase in Ca^2+^ concentration in Figure 5b, the temperature and shear resistance of its gel gradually weakened, and when the CaCl_2_ concentration was higher than 1500 mg/L, the viscosity decreased below 50 mPa·s after shearing for 50 min at 80 °C. CaCl_2_ was the most influential component in fracturing processes, and it decreased the viscosity and disturbed the fluid system equilibrium in the presence of high concentrations of Ca^2+^. When CaCl_2_ was added, in the alkaline fracturing fluid environment, a large amount of OH^−^ combined with calcium ions to form Ca(OH)_2_, which is slightly soluble in water, and a large number of flocculent precipitates appeared, forming charged colloids. The presence of these ions could delay or prevent polymer hydration, rendering it difficult to swell or dissolve in water [9,19]. Therefore, with the increase in Ca^2+^, the obstruction of polymer extension became more obvious, and its temperature and shear resistance properties became worse.

#### 2.2.3. Effect of FeCl_3_ Content on the Shear Performance of Fracturing Fluid

**Figure 6:**
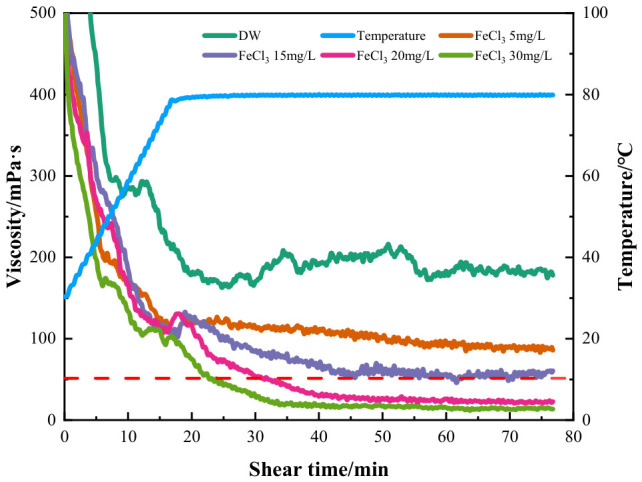
Rheology of HPG fracturing fluid prepared with different concentrations of FeCl_3_.

Although the common trivalent iron concentration in seawater is very low, the used water always contains iron ions due to corrosion from exploitation equipment and pipes. It can have a significant effect on the rheology of fracturing fluids. Therefore, experiments at Fe^3+^ below 30 mg/L were also performed, and the results are shown in Figure 6. As the FeCl_3_ concentration increased, the viscosity of fracturing fluid showed a monotonic decreasing trend, dropping from nearly 500 mPa·s to approximately 20 mPa·s at 30 mg/L Fe^3+^. When a very small amount of trivalent iron ion was present (5 mg/L), the fluid’s viscosity decreased distinctly compared with the blank control sample. With increasing Fe^3+^ ion concentration, the viscosity of the fracturing fluid decreased rapidly, and when the concentration was higher than 20 mg/L, only the 30 mg/L Fe^3+^ system dropped below 50 mPa·s within 30 min, while the 20 mg/L sample remained above 50 mPa·s. The fast viscosity decline induced by Fe^3+^ ions was caused by insufficient crosslinking of the fluid, and prolonged shear also deteriorated the viscosity. It is probably because the precipitation of Fe^3+^ and its coordination effect with hydroxyl groups reduced the electrostatic repulsion between charges in the polymer backbone. This formed an electrostatic shielding effect, inhibited the stretching of HPG thickener molecular chains, and reduced the viscosity and shear resistance of the fracturing fluid system [35,36]. It was seen that among the cations, Fe^3+^ was the most influential in fracturing processes, and the fluid containing Fe^3+^ could not meet the fracturing requirements of field applications under untreated conditions [37].

### 2.3. Dynamic Viscoelasticity Analysis

**Figure 7:**
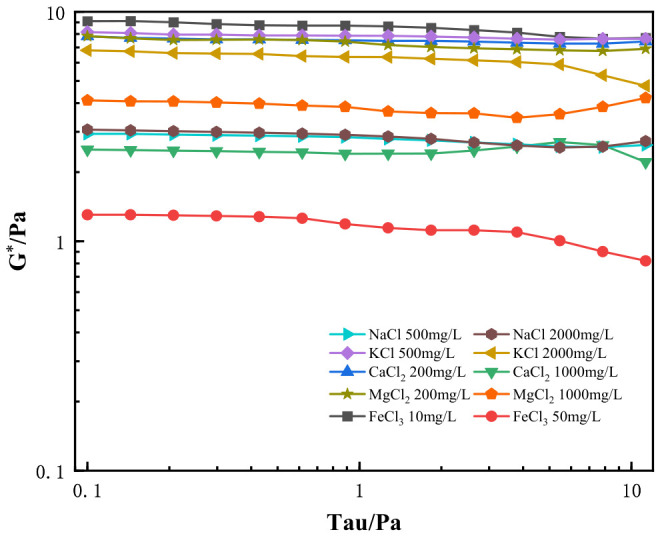
Shear Stress vs. Complex Modulus (G*) for HPG Gel with Different Ions.

The linear viscoelastic region of HPG gel is determined by the complex modulus (G*) and shear stress, which agrees well with the fundamental linear viscoelastic theory of borate-crosslinked guar gum gels reported in Ref. [38]. As shown in Figure 7, the gel was tested under a shear stress range of 0.1–10 Pa. At lower shear stresses, the stress of the gel was proportional to the strain, during which the complex modulus of gels with different ions remained basically stable. The deformation was dominated by elasticity (G′), and energy was primarily stored, which is consistent with the stable linear viscoelastic response of inorganic ion-containing HPG fracturing fluids demonstrated in Ref. [39]. As the shear stress increased, 2000 mg/L KCl, 1000 mg/L CaCl_2_, and 50 mg/L FeCl_3_ exhibited clear yield points within the tested range, causing G* to begin to decline. The elastic characteristics weakened, and energy consumption increased. However, gels prepared with other ions did not show such behavior within this range, indicating that they remained within the linear viscoelastic region of the prepared gels. Finally, we chose to conduct frequency scans at shear stresses corresponding to the tau values of 0.8 Pa, 1 Pa, 1 Pa, 0.7 Pa, and 0.5 Pa for NaCl, KCl, CaCl_2_, MgCl_2_, and FeCl_3_, respectively.

#### 2.3.1. Effect of Monovalent Ions on the Dynamic Viscoelasticity of HPG Gels

Viscoelasticity is one of the most important parameters for assessing the suspension capacity of fracturing fluid proppant [40]. It is generally believed that conventional guar gum gel fracturing fluid relies on viscous sand-carrying, while surfactant fracturing fluid relies on elastic sand-carrying. Viscosity does not fully reflect their performance for the gels used as fracturing fluids [30]. Since the degree of frequency dependence of elastic modulus (G′) can be considered as an indication of the viscoelastic nature of gels, the sand-carrying of fracturing fluid relies on the dual effects of viscosity and elasticity (the viscosity of fracturing fluids is usually not <50 mPa·s, G′ ≥ 1.5 Pa, G″ ≥ 0.3 Pa). The elastic resistance of viscoelastic fluids is mainly related to the G′ and G″ of the viscoelastic fluid and the zero-cut viscosity (η0), and the larger the ratio of G′/G″, the higher the elastic resistance possessed by the fluid, the more favorable it is to inhibit the sinking of proppant and assure the proppant-carrying capacity. Thus, the effect of salts on the properties of the gels was further investigated using dynamic rheological data with the complex shear modulus (storage modulus, G′, and loss modulus, G″).

**Figure 8:**
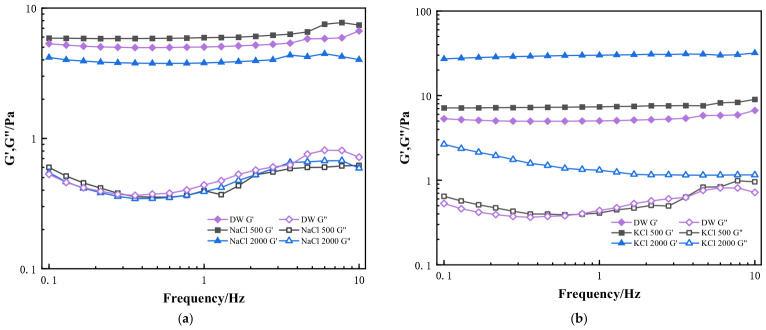
Storage modulus (G′, solid lines) and loss modulus (G″, dashed lines) of guar gum at 20 °C and frequency of 0.1~10 Hz. (**a**) Different NaCl concentrations; (**b**) Different KCl concentrations (unit: mg·L^−1^).

Figure 8a and Figure 8b show the viscoelasticity of the HPG gels containing different concentrations of NaCl and KCl, respectively. The storage (G′, solid lines) and loss moduli (G″, dashed lines) changes in HPG gels (20 °C, f = 0.1~10 Hz) in 500 mg/L, 2000 mg/L KCl or NaCl solution and deionized water were also recorded. It was seen that the linear viscoelastic range of the gels containing Na^+^ and K^+^ was wide, and G′ > G″ in the frequency range of 0.1–10 Hz. When the KCl and NaCl concentrations were more than 2000 mg/L, the viscoelastic profiles showed G′ > 1.5 Pa and G″ > 0.3 Pa within 0.1–10 Hz. For the NaCl and KCl systems, the G′ values for the guar gels were significantly higher than the G″ values of guar gels, indicating that the elastic resistance possessed by the fluid in NaCl and KCl solutions was larger [41]. It was more favorable to inhibit the sinking of proppant and ensure the proppant-carrying capacity. Different from the NaCl system, the elastic modulus G′ at a KCl concentration of 2000 mg/L was higher than that at a lower concentration, and the G′/G″ value was higher. This behavior suggests that, due to the larger size and higher charge of K^+^ ions, the interactions between K^+^ ions and water molecules are stronger than those between Na^+^ ions. In high-concentration solutions, the strong hydration of K^+^ ions leads to a more tightly bound gel structure, forming an interconnected gel-like network dominated by elastic behavior. As a result, the gel strength (G′) of guar gum remains the highest [42].

#### 2.3.2. Effect of Divalent Ions on the Dynamic Viscoelasticity of HPG Gels

**Figure 9:**
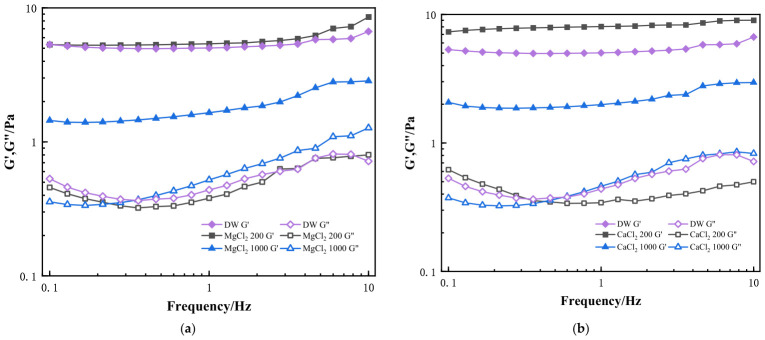
Storage modulus (G′, solid lines) and loss modulus (G″, dashed lines) of guar gum at 20 °C, frequency range of 0.1–10 Hz. (**a**) Different MgCl_2_ concentrations; (**b**) Different CaCl_2_ concentrations (unit: mg·L^−1^).

Figure 9a,b shows the viscoelasticity of the HPG gels containing different concentrations of MgCl_2_ and CaCl_2_. The storage (G′, solid lines) and loss moduli (G″, dashed lines) changes in HPG gels (20 °C, f = 0.1~10 Hz) in 500 mg/L, 2000 mg/L MgCl_2_, or CaCl_2_ solution and deionized water were recorded, respectively. When the MgCl_2_ and CaCl_2_ concentrations were 500 mg/L, the viscoelastic profiles showed that the G′ and G″ values did not decrease and were higher than those in deionized water. Moreover, G′ was always higher than G″. It indicates that elasticity was the dominant gel behavior. However, when MgCl_2_ and CaCl_2_ concentrations increased to 2000 mg/L, the G′ values obviously decreased, and the elastic resistance possessed by the fluid was lower. It was shown that the weaker gel structure developed at higher Ca^2+^ and Mg^2+^ concentrations. Therefore, the effect of divalent ions was unfavorable for inhibiting the sinking of proppant and ensuring the proppant-carrying capacity in MgCl_2_ and CaCl_2_ fluids [43]. We also found that the effect of CaCl_2_ on the viscoelasticity of fracturing fluid was relatively large, especially when calcium ions were at 200 mg/L, the values of G′/G″ became lower, and the elastic resistance possessed by the fluid decreased, which was not conducive to inhibiting the sinking of proppant [44,45]. It was different at the same low concentration of MgCl_2_.

#### 2.3.3. Effect of Trivalent Ions on the Dynamic Viscoelasticity of HPG Gels

**Figure 10:**
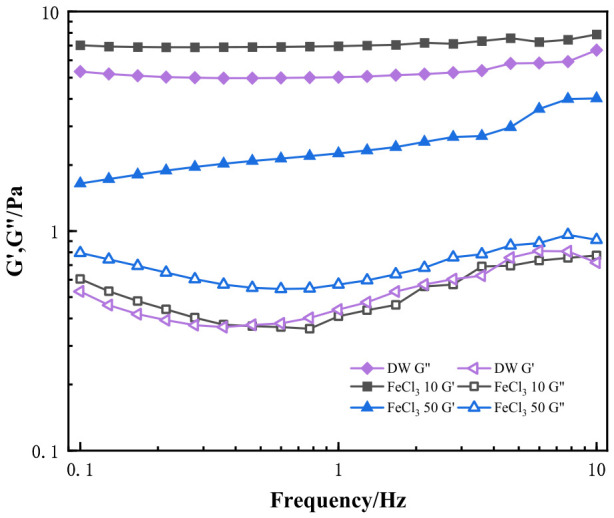
Storage (G′, solid lines) and loss moduli (G″, dashed lines) changes in guar gum (20 °C, f = 0.1~10 Hz) at different FeCl_3_ concentrations (in mg·L^−1^).

In Figure 10, the storage (G′, solid lines) and loss moduli (G″, dashed lines) changes in HPG gels (20 °C, f = 0.1~10 Hz) in 10 and 50 mg/L FeCl_3_ solution and deionized water are recorded, respectively. Similarly, the G′ and G″ values in the CaCl_2_ system were higher than those in deionized water in a 10 mg/L FeCl_3_ solution, and G′ was always higher than G″. The G′ value of the FeCl_3_ system decreased from 10 mg/L to 50 mg/L, and the G′/G″ ratio became smaller, indicating that a weaker gel structure formed at higher iron concentrations, and a lower structural strength (G′) of guar gum gels was achieved. This behavior reflects that the interconnected gel-like network structures with mainly elastic behavior were destroyed because of complexation and electrostatic repulsion of trivalent ions. Therefore, a small amount of iron ions has a significant effect on the viscoelasticity of fracturing fluid gel, which would affect the application of fracturing fluids [46,47].

**Figure 11:**
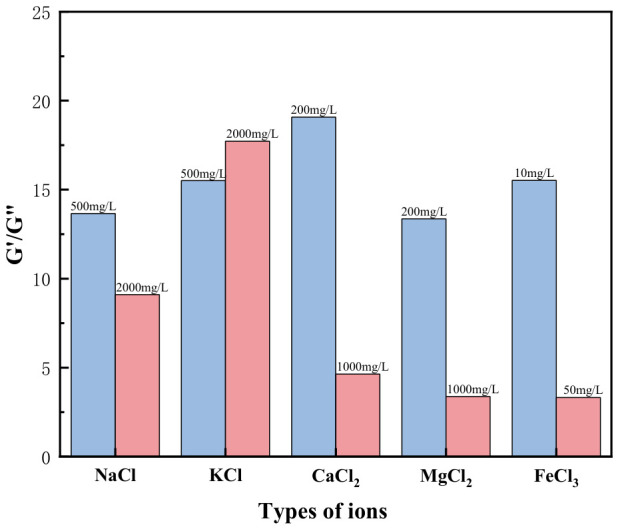
Effect of Ions on the G′/G″ Ratio in HPG Gel.

Figure 11 shows the effect of different ions on the G′/G″ ratio in HPG gel. As the concentration increases, for monovalent ions, the G′ value of guar gum remains significantly higher than the G″ value, indicating better elastic resistance, which helps reduce proppant settling. This is because, as the ion concentration increases, hydration and ionization effects are enhanced, promoting intermolecular crosslinking or aggregation, resulting in a more compact network structure. In contrast, under the influence of Ca^2+^, Mg^2+^, and Fe^3+^, the elastic modulus (G′) of the guar gum decreases while the viscous modulus (G″) increases. This causes the G′/G″ ratio to decrease significantly, which greatly affects the viscoelasticity of the gel. This is due to the chelation effect of the ions, which disrupts the gel’s network structure. As the ion concentration increases, the interactions between ions intensify, leading to electrostatic shielding, which reduces the cohesive forces between the solute molecules.

### 2.4. Microstructure Analysis

#### 2.4.1. SEM Analysis of Fracturing Fluid Gel Including Different Salt Compositions

**Figure 12:**
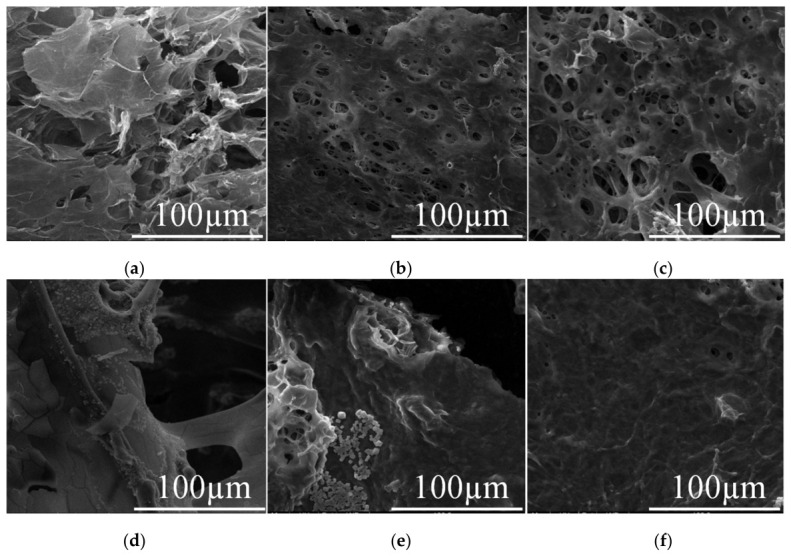
SEM images for the fracturing fluid containing different salt compositions: (**a**) deionized water, (**b**) 2000 mg/L NaCl, (**c**) 2000 mg/L KCl, (**d**) 1000 mg/L MgCl_2_, (**e**) 1000 mg/L CaCl_2_, (**f**) 30 mg/L FeCl_3_.

SEM was used to examine the microstructure of HPG gel formulated with different brines (Figure 12). Since conventional SEM does not allow for the determination of samples containing water, the low-temperature vacuum freeze-drying technique was used to avoid damaging the microstructure. As shown in Figure 12, the appearance of many microstructures between crosslinked HPG gel molecules was quite different when using different types of brines. In deionized water, the HPG fibers appeared connected with one another to form a multilayered sheet-like structure, as shown in Figure 12a. With the addition of 2000 mg/L NaCl or KCl, the degree of crosslinking between HPG molecules increased, the HPG fibers were entangled and strongly interpenetrated, and network structures were formed, as shown in Figure 12b,c. Moreover, void spaces in a net-like formation, forming a relatively dense 3D network structure, were observed among the fibers, including KCl, which indicated that the elasticity of the gel was stronger than that of NaCl. Although the effect of the magnesium ions on the viscoelasticity of gels was relatively small and their viscoelastic curve was relatively stable, the polymer containing MgCl_2_ spread into a thin network structure, as shown in Figure 12d. However, the crosslinked structure of gel was destroyed when 1000 mg/L CaCl_2_ and 30 mg/L FeCl_3_ were present, which did not show the network structure and became a hard gel, as shown in Figure 12e,f, indicating that Ca^2+^ and Fe^3+^ ions could delay or prevent polymer hydration and reduce gel stability, and the formed gel became denser and gelatinous. This is consistent with the conclusion of the rheological effect of ions on fracturing fluids.

#### 2.4.2. Salts’ Effect on Gel Properties

**Figure 13:**
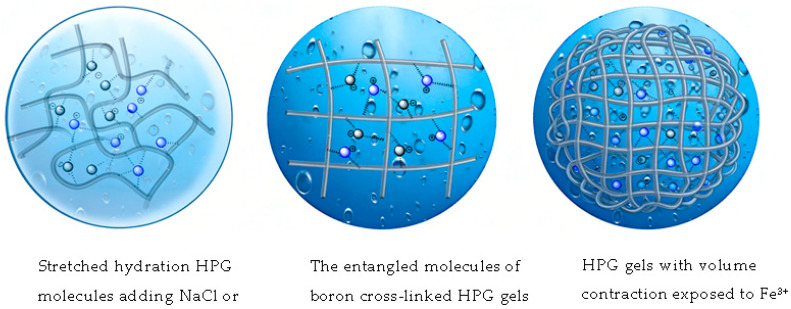
Schematic diagram of the crosslinked fracturing fluid gel structure with different ions.

The properties of the solution are completely determined by the interaction between the molecules of lyophilized gum and solvent water molecules. The molecules of HPG gum show long-chain distribution, and the long chains are entangled with each other through the crosslinking of boron to form irregular network structures with different scales of cavities, and many polymer molecular groups interact with each other and curl to form an aggregated state with tight distribution and small spacing, which macroscopically shows high viscosity and high elasticity [22,48,49]. As NaCl and KCl are added, as shown in Figure 13, the intermolecular covalent bonds and hydrogen bonding are broken to a certain extent due to the effect of ionic charge. Thus, the curled polymer molecules are stretched, and hydration is enhanced, and the shear viscosity is not reduced or even slightly increased [50]. When high-valence metal ions are added, as shown in Figure 13, HPG gels undergo volume contraction when exposed to Fe^3+^ salt. This phenomenon occurs due to the interaction between the Fe^3+^ and the gel matrix, which leads to a reduction in the space occupied by the gel network. The specific mechanism involves the screening of electrostatic repulsions between the charged groups within the gel by Fe^3+^, as well as coordination of Fe^3+^, resulting in a more compact structure and, consequently, a decrease in volume. This effect is often utilized in various applications to control the rheological properties of the gels [51].

## 3. Conclusions

This study systematically evaluated the differential effects of common inorganic ions on the rheological performance of borate-crosslinked hydroxypropyl guar fracturing fluids, providing both mechanistic insights and practical guidance for the use of saline water in hydraulic fracturing. The key findings are as follows: Monovalent ions (Na^+^, K^+^) and SO_4_^2−^ have minimal influence on fluid viscosity and viscoelasticity, even at concentrations exceeding 10,000 mg/L. These ions promote polymer hydration and network formation, maintaining G′ > 1.5 Pa and viscosity above 100 mPa·s under shear at 80 °C. Divalent Mg^2+^ exhibits moderate effects, with viscosity remaining above 100 mPa·s up to 2500 mg/L. However, Ca^2+^ significantly degrades fluid performance at concentrations >1500 mg/L, reducing viscosity below 50 mPa·s and decreasing the G′/G″ ratio, indicating weakened gel elasticity and proppant suspension capacity. Trivalent Fe^3+^ is the most detrimental ion: concentrations as low as 15 mg/L cause rapid viscosity loss to <50 mPa·s at 80 °C and 170 s^−1^, rendering the fluid unsuitable for fracturing operations. This effect arises from Fe^3+^-induced complexation and electrostatic repulsion that disrupts the borate-HPG crosslinking network. Microstructural evidence corroborates rheological findings: Na^+^ and K^+^ promote dense, entangled HPG networks, while Ca^2+^ and Fe^3+^ destroy the crosslinked structure, forming compacted, non-porous gel aggregates.

These results establish a clear ion-specific hierarchy of influence on HPG gel rheology and demonstrate that effective fracturing fluid formulations can be prepared using high-salinity water sources, provided that problematic ions (particularly Ca^2+^ and Fe^3+^) are controlled or removed. The findings support sustainable fracturing operations by enabling targeted pretreatment strategies for produced and flowback waters, reducing freshwater consumption without compromising fluid performance.

## 4. Experimental Materials and Methods

### 4.1. Materials and Reagents

#### 4.1.1. Preparation of Simulated Saltwater

The main reagents are industrial products, including HPG, borax (Na_2_B_4_O_7_·10H_2_O), ammonium persulfate, NaOH, NaCl, KCl, Na_2_SO_4_, CaCl_2_, MgCl_2_·6H_2_O, and FeCl_3,_ all of which were analytically pure. HPG was kindly supplied by Henan Alpha Chemical Co. The molecular weight of HPG is 2.2 × 10^5^. NaOH, NaCl, KCl, Na_2_SO_4_, CaCl_2_, MgCl_2_, FeCl_3,_ and Na_2_B_4_O_7_·10H_2_O used in this study were of assay grade reagents (Sinopharm Chemical Reagent Co. Ltd., Shanghai, China) and were used without further purification.

Table 1 lists the analysis results of flowback water in northwestern China. Based on the water composition, the experimental saline water was prepared using specific concentrations of NaCl, KCl, Na_2_SO_4_, MgCl_2_, and FeCl_3_. The detailed method was as follows: 0.1385 g, 0.4155 g, 0.6923 g, 1.3846 g, 2.0768 g, 2.7691 g of anhydrous CaCl_2_ were weighed on an analytical balance and dissolved in 500 mL of deionized water, resulting in Ca^2+^ concentrations of 100 mg/L, 300 mg/L, 500 mg/L, 1000 mg/L, 1500 mg/L, 2000 mg/L in the saline solution.

#### 4.1.2. Preparation of Base Fluid

Powders of HPG were dispersed in simulated saltwater and stirred at high speed for 5 min on a GJ-3S high-speed stirrer (Qingdao Haitongda Special Instrument Co., Ltd., Qingdao, Shandong, China) to form a homogeneous solution, which was then swelled at 30 °C for 4 h. The base fluid containing 0.35% HPG was prepared [52].

#### 4.1.3. Preparation of HPG Fracturing Fluid

A stock solution of HPG was mixed with the borax crosslinker and stirred until a gel formed at room temperature (25 °C). For all samples containing salts, the base fluid in saline water was first prepared, and then the HPG solution was mixed with borax, with gentle stirring at room temperature. In this study, the addition of the crosslinker was 0.4% in all crosslinked gels.

### 4.2. Testing and Characterization Methods

#### 4.2.1. Rheological Measurement of Fracturing Fluid

All control experiments and repeatability tests for rheological measurements were carried out under the same experimental conditions as described below, ensuring the reliability and repeatability of the test data.

The steady-state rheological measurements were performed using a HAAKE RS-300 rotational rheometer (Thermo Fisher Scientific, Waltham, MA, USA) with a closed concentric cylinder. The apparent viscosity of the gels was obtained by shearing at 80 °C at a constant shear rate of 170 s^−1^ for 10 min. The heating rate was controlled from 30 °C to 80 °C at a rate of (3 ± 0.2) °C/min. After reaching the test temperature, the gel was kept at a constant temperature and sheared for more than 60 min [53].

An Anton Paar rheometer (MCR302, Anton Paar GmbH, Graz, Austria) with PP50-SN31020 plate fixture (gap = 1 mm) was used to perform stress-strain scanning to observe the variation in the composite modulus (G*) with oscillatory stress to determine the linear viscoelastic region of the fracturing fluid [49]. Firstly, the stress sweep experiments at the frequency of 1 Hz were conducted from 0.01 to 10 Pa to determine the linear viscoelastic region. Secondly, frequency sweeps were conducted over the range of 0.1–10 Hz under selected stress within the linear viscoelastic region. The oscillatory stress was fixed, and the energy storage modulus (G′) and loss modulus (G″) of the fracturing fluid were determined to get insight into the network structure and viscoelastic behavior of multi-composite gels.

#### 4.2.2. Scanning Electron Microscopy (SEM)

A scanning electron microscope (Quanta 450, FEI Company, Hillsboro, OR, USA) was used for imaging the fracture surface of crosslinked HPG gels [54]. The prepared samples for SEM analysis were frozen in liquid nitrogen and dried under vacuum in an FC-27AS-E vacuum freeze dryer (Tokyo Rikakikai Co., Ltd., Tokyo, Japan). Then, the solid samples were sprayed with gold powder on the surface, and the microscopic morphology of the gel samples was observed by a JSM-6700 field emission scanning electron microscope (JEOL Ltd., Tokyo, Japan) in high vacuum mode at 20 kV and 3.5 nm resolution.

## Figures and Tables

**Table 1:** Analysis of ions in fracturing flowback fluid in Yanchang Oilfield.

Ions	Concentration (mg/L)
K^+^/Na^+^	39,369
Ca^2+^	3157
Mg^2+^	868
Ca^2+^ and Mg^2+^	4025
Ba^2+^ and Sr^2+^	179
Fe^3+^	13
Cl^−^	69,471
Br^−^	187
HCO_3_^−^	188
CO_3_^2−^	41
SO_4_^2−^	751
total mineralization	118,249
Type of water	CaCl_2_

## Data Availability

The original contributions presented in this study are included in the article. Further inquiries can be directed to the corresponding author(s).
